# Sister Olive: A Hospital Romance

**Published:** 1886-12-11

**Authors:** W. J. Eccott


### Sister Olive: A Hospital Romance.

By W. J. Eccott.

### CHAPTER II.

Some days have passed since that interview. Hamelin Grant had gone about with a heavy heart and very little will for work. His time hung the heavier that it was by no means so fully occupied as it had been, while he was house physician to the Infirmary. He and Halsden, his old classmate and greatest friend, were going to try their luck together in practice. They had furnished a small house in a respectable street. Both were men of some private means. Both had been much liked by the patients at the Infirmary. In a thickly populated town like Cottingham, busy with mills and factories, seething with work-people, and thronged on its outskirts with wealthy men of business who were given to excessive high-living and various other, by them deemed fashionable, ways; if there wasn't work for two bright young doctors fresh from the most modern school of professional experience, for whom would there be room?

Meanwhile Grant put in a good deal of time at the Infirmary; casually dropping in and looking about here and there, chatting to the nurses, watching cases and giving a hand where necessary. He told Halsden he wanted to keep his hand in and especially to watch that enteric fever case. Certainly he had taken an interest in that from the outset, and his love for every detail of his profession was undisputed. One thing he wouldn't own to himself—he would indeed have been as ready to confess that his mind was losing its balance—that he ever went to the Infirmary to be near, to see, or to hear Sister Olive. Halsden, who knew his friend well, noticed Grant's sad face and guessed the reason, but, like the good fellow he was, said nothing nor evinced any dissonant jocularity, which would only have jarred on Grant's feelings.

Grant noticed during those first few days of his practice that the case of John Hargen, the "enteric fever case" as it was called, was interesting Sister Olive very much. There seemed to be more than her usual solicitude displayed: more anxiety. The man was coming round gradually. All delirium had ceased, and though he was very weak, he was conscious. And whenever Sister Olive was about the ward, it was wonderful to see how that man watched her. There was a wistful look of dog-like affection upon his face, and a strongly marked face it was too—the face of a man whose life wheels had been driven furiously: the traces were there in the long furrows of his forehead and upon his lips. Two other facts Grant noticed. The one was the extreme coldness that existed between Bernard Lumley, the new physician, and Sister Olive, a feeling which seemed to be mutual. The other fact was the wild gleam that came into Hargen's eyes whenever he saw Lumley.

Hamelin Grant was surprised on coming in one day, after a few days' absence, to find that John Hargen was gone. Dr. Lumley, so a nurse told him, had expressed his opinion that Hargen was quite well enough to leave the hospital and especially as the bed was wanted. He also heard that there had been a heated discussion between Sister Olive and the new house-physician on the subject.

"Well," said Grant to himself, "if that doesn't kill that man Hargen off, he's got a wonderful constitution." "By the way, nurse," he said aloud, "Do you know what his address is—his lodgings?" "Sister Olive knows, Sir. I'll go and ask"; and presently the nurse came back—"17, Long Row, third floor, Sir."

In a few minutes, after calling in at the Dispensary for some medicine, Grant started off for Long Row. It was in rather a low-lying part of the city, not more than seven minutes' walk from the Infirmary, and only a few yards from a canal. It was a dingy street into which little sun ever penetrated by reason of the shade cast by the many huge warehouses on either side. The few dwelling-houses that alternated now and then with the other buildings, were generally big tenement houses above small shops, houses where almost every room was occupied by a separate family, and every family made use of the roadway as a common receptacle for filth and garbage by the common process of throwing such from their respective windows: houses where families washed, if they ever did use clean linen, at home, and cooked and slept and even took in lodgers in the same apartment. Over all the house permeated as a matter of course a steamy atmosphere, redolent of mysterious odours of stale viands.

Some tangled-haired barefooted children were playing around the doorway of No. 17. Grant asked one if she knew of a Mr. Hargen. The child looked at him somewhat composedly, probably debating within herself whether to class him as a "School Board man" or not, and then ran into the house shouting "Mother!" Meanwhile the other children stood in front of him close together and surveyed him as if he was a curiosity.

"Mother" made her appearance with bare arms, from which she was hastily wiping steaming soapsuds. Recognising Doctor Grant as one of the "gents" from the "Infirmary" she curtsied and said:—

"Who did you want, sir? Come to see our Jim? Jim's at work now at Maxwell's 'long with his father."

"No! I didn't come for Jim. I wished to know if anyone named Hargen lived here?"

"'Argen? 'Argen? No I can't say as how I knows the name, "But there's Mrs. Jupp as lives up on the third floor. She's got a lodger. I cert'nly did hear as he was from the 'Firmary. Ring the top bell there, sir, and go up!"

Then, as Grant complied, the matron went up a few stairs and delivered a loud shrill "Missus Ju-upp?" When Grant had mounted, he was soon ushered by a talkative woman, who announced herself as Mrs. Jupp, into a desolate-looking small room which looked out over dense volumes of smoke and a collection of roofs and chimney-stacks of various shapes and sizes agreeing in little else than their extreme dingy ugliness. Over the fire, for there was a fire, was crouched the fever patient. His eyes lit up when he saw who it was, and he said with a good accent:

"Take a seat, Doctor! Not much choice. I'm sure its very kind of you to look me up. I'm sorry the apartments are not so palatial as they should be." And then his grim sardonic smile relapsed into a long-drawn expression of hopelessness.

"Well, how are you by this time?" said Grant, sitting down on the edge of the bed and looking at him keenly.

The man described his condition bit by bit, some parts of his account being skilfully drawn out by a question or two of Grant's. He was as weak as it was possible to be, and yet be out of bed.

"You ought to have been in the hospital for another week at least to get up your strength. How is it possible to fight against disease and keep it out in your condition, and in a place like this? Why, if it had been a plot it could not have been better calculated to finish you."

"Ah! I should have stayed, if it hadn't been for that cursed Lumley," said the man between his teeth and with a scowl upon his face. "All his doings, I know. Did it on purpose I shouldn't wonder. There was Olive did all she could, I know, but she couldn't manage it. It isn't the first time, Lumley, curse you! Ugh! its cold," Hargen shivered. "Not much like our last passage up the Suez Canal. Kept warm there!"

Grant would have liked to ask questions about Lumley, but desisted and said:

"You've travelled?"

"Yes! all the world over pretty nearly. Pillar to post, post to pillar, regular rolling stone. Never gathered moss nor money. Always turned up home at the wrong time. Cold shoulder of course. My own fault partly, I suppose. Seemed hard, tho'. I've had chances and lost them and ruined other people's. But, by——," and here he clenched his fist, "if I can only pick up strength somehow. This last turn's done something for me, besides nearly doing for me altogether; it's made me see something I've been knocking my head against for years and never saw. And the rest of my life's going to be some sort of apology for it. Can you get me anything to do? I'm an educated man, and there's little I can't turn my hand to."

"To do, Hargen! The first thing's to get well; and as soon as I can hear of decent lodgings anywhere, you must move."

"Move!" Hargen interposed bitterly. "My next move will be to the workhouse, I'm thinking, or to that corner of the cemetery where the parochial authorities don't consider it form to put a memorial stone."

"Don't talk like that, man!" said Grant, cheerily. "Only pick up your strength, and there's plenty of use for you yet."

"Well! and where am I to find the food to get stronger? Or the money to pay for decent lodgings?"

"Oh! I'll look after that," said Grant, quietly. "I'm going to see you through this."

The patient looked at him with a curious stare, and then said, "Then there are two or three good fellows left in the world after all, and I'd just made up my mind the world was nothing but a mass of selfishness."

Grant now produced the medicine and placed it on the mantel-piece. "There! take that according to directions. I'll send you down some beef-tea. It's no use trusting Mrs. Jupp to make it. There isn't one woman in twenty can make beef-tea. You can heat it yourself over your fire, and I'll look in again to-morrow. I'll send you down a book to read, and all you've got to do is to keep your spirits up as much as possible."

---

### CHAPTER III.

As Grant turned into the street again and looked up and down an instant before deciding which way to go, he caught sight of a familiar figure coming down the street. He took the other way, and, as he neared the bottom, casually turned round and saw it disappear into the very house he had just left. It was Sister Olive. And this set him off thinking till he reached home.

That evening, as Halsden and he sat after dinner before the fire, Halsden reading, Grant deceiving himself that he was doing the same, his thoughts were wandering away to Olive and Hargen. He was looking very sad and thoughtful. Finally he closed the book, took down his pipe from the mantel, filled it, and began smoking.

Halsden looked up from his book:

"Smoking so soon?" he asked.

"Yes!" replied Grant—"I can't read. Something's come over me lately. I've had no humour for reading."

Halsden looked from under his shaggy eyebrows out of his deep-set eyes with a sympathetic, tender glance, and just a slight smile seemed to play about his lips.

Then he took his pipe from its case and set to smoking, and both puffed away some time in silence.

"Have you ever felt, Halsden," at length Grant said, "as if you were living by clockwork? Going through every part of the day as if you were a machine, and the other part, the thinking self, was not there at all—was ceaselessly worrying over the same subject, revolving the same series of mind-pictures over and over again, the first thing on waking in the morning, the last thing at night? Do you understand what I mean?"

Halsden took the pipe from his mouth and knocked out some of the ashes. "Well! Yes! Something of the sort I remember, when I was first smitten with the love fever. I didn't get much sleep then for a few weeks, always wondering whether I'd got a chance. There were more fellows than I on the war-path. I was always trying to glean some sort of sunshine out of a few casual words of hers, always setting this against that, and coming to the conclusion I hadn't got any chance, and then coming back to it again. No; I didn't get much work done then. But, by Jove! she's been a guardian angel since. Many's the spree I haven't gone to with the other men, just by thinking of how she would take it if she knew; many the hard grind I've done because I felt I've been working for her and not merely for ambition."

It was not long before Halsden drew out of his friend what was the cause of his unhappiness, and, once that confession made, Grant soon laid bare most of his thoughts on the subject.

"You don't think," he said, "that Archie Tenison is at the bottom of it—in fact, has in reality a prior claim to her affection, one from which she is too honourable to draw back?"

"Well, of course I don't know Sister Olive well," returned Halsden. "But look at Archie Tenison and look at you. Archie's a sharp fellow, coming on well in his exams., but he's another year yet before he gets through. You are a man of already five years' experience. From what I've seen of Sister Olive, I should say she is no girl, I don't mean to say she's old, but she bears that in her face and manner which tells of some pretty considerable experiences, which must have moulded her mind to a different groove from Archie Tenison's. In fact, he must still be a boy to her. Women of her cast marry men, not boys. No; there's something else in it."

"What can it be, then?" said Grant. "From what she said, I guessed that there was something in her history which rightly, or wrongly, she wishes to keep secret, some family skeleton which would subject her, if exposed, to a malign interpretation in the world's mouth. Now, there's that Bernard Lumley, the new physician. From something she let drop, she's no friend of his. Then there's Hargen: I'm tolerably sure he knows Lumley too, And Olive showed great interest in Hargen. There's something behind the scenes there."

"By the way," Halsden exclaimed, "I knew I'd got something important to tell you, and your mention of Lumley recalled it. I met Leslie, the student, to-day, and he says that Sister Olive's leaving the Infirmary."

"Leaving the Infirmary!" Grant slowly repeated. "Surely not leaving!"

"Yes; leaving!"

"And the reason?"

"He doesn't know. He thinks that it is partly owing to friction in hospital work between Lumley and her. But he also thinks there's some private quarrel, for, on one occasion he overheard Sister Olive's voice speaking in angry, indignant tones, very unlike her usual self; of course Leslie drew back and left. Depend upon it, they've met before; and if, as you say, that man Hargen knows Lumley, there lies the key. Tackle Hargen, and find out all you can."

Grant was much perturbed by the news Halsden had communicated. As long as Sister Olive was at the Infirmary she was within sight. Now that she was leaving, if she had not already left, he felt that he would be more uneasy than he had been. That she would leave Cottingham, no longer having any object to keep her there, he readily made up his mind. Should he, he asked himself, seek another interview? Such a course would only pain her, his mind answered. If she loved him, surely she would at least let him know her next address. But did she love him? He would fain have flattered himself that she did. But he was diffident of the impression he had made. Were her utterances in that interview merely those of sympathy with his case, of genuine admiration for his character, but as far apart from the feeling of love as one pole from the other? His self-questionings were severe on this point, but he would not suffer himself to gather aught of hope from them. The wish was father to the thought, and as such the thought must be looked upon with suspicion. But she had said, "You will always be a dear friend!" From his knowledge of the depth of her character, from the tones in which she had uttered them, Grant was assured that those words were no empty phrasing, but the expression of a real feeling. In them hope lay concealed, a veritable seedling now, but one which would burst forth in a fairer future. "Yes!" he told himself, "she will write."

But two or three days came and went, and no word reached him. The hope became fainter, but it was there in his mind firmly imbedded. He went about his work cheerily enough. Cases were coming in more plentifully than he could reasonably have expected: poor cases all, but still they meant practice, and full employment for his thoughts. Not that he forgot Hargen by any means. He had been true to his word, and looked about. After two or three visits he was able to announce to him that he had found something better for him in the way of lodgings. The next day saw Hargen installed in them. It was a cottage on the very outskirts of the town, on the road to the suburb of Fothercliffe, where many of the merchant princelets and millionaires of Cottingham had raised their villas and laid down their tennis-lawns. From the dormer window upstairs could be seen a long stretch of real country —hill, green field, and woodland, and not so much as a builder's notice board of "Desirable Building Lots" to interrupt the view. In the commodious chamber possessing this welcome look-out Hargen took up his next abode. The room was clean and comfortable. The landlady was one of those round, smiling, good-natured bodies whose very aspect is a curative, and whose cheery tones and bustling motherly ways are often of more avail than a week at the seaside.

It was the evening after Hargen's removal that Grant walked out to Petunia Cottage; Halsden said he should be in all the evening, and told Grant, in his pleasant way, that he would take care not to poison anybody before his return, and that, if patients should happen to flock in too fast, he would send a message.

Grant knocked at the door, but Mrs. Hussey, the landlady, had evidently heard him fumbling at the garden wicket, for his finger had scarcely touched the knocker before the door opened, and a glow of cheery light from within shone on the pathway, while she herself stood revealed in the entrance.

"Good evening, doctor; come in, sir. You'll find Mr. Hargen upstairs. Stay a minute, sir, and I'll get a light." She bustled into the little room, half kitchen, half sitting-room, where her husband sat before the fire smoking, and reached a benzoline lamp down from the mantel, and bustled out again in a few seconds. Then she resumed: "He do look poorly, sir. But he's brighter to-day than he was yesterday. Lor! Fever do pull a body down, sir, don't it? This way, sir," and she tripped up the steep, narrow stairway, turned along the little landing, and opened a door. Grant thanked her and entered.

It was a snug room with a sloping ceiling. There was a nice easy-chair and a little table covered with a bright cloth. The sheets on the bed were snowy white; the patchwork counterpane was clean as could be. The great knobs at the corners of the old-fashioned wooden bedstead shone with the lustre imparted by twenty years' application of unstinted labour. There was a good fire in the grate, and Hargen, with a pillow behind him, was comfortably seated reading by the light of a lamp. A few grapes from his landlord's hot-house (he was a market gardener) lay on a plate before him. Seeing Grant, he was rising.

"Don't move; keep comfortable. I'll find a seat," said Grant, and drew up another chair opposite. "Now, then, how are you?"

*(To be continued.)*

